# Synergistic effects of mutation and glycosylation on disease progression

**DOI:** 10.3389/fmolb.2025.1550815

**Published:** 2025-02-04

**Authors:** Shodai Suzuki, Motoyuki Itoh

**Affiliations:** ^1^ Department of Biochemistry, Graduate School of Pharmaceutical Sciences, Chiba University, Chiba, Japan; ^2^ Research Institute of Disaster Medicine, Chiba University, Chiba, Japan; ^3^ Health and Disease Omics Center, Chiba University, Chiba, Japan

**Keywords:** glycosylation, mutation, aging, Notch3, LDLR, APP, CADASIL, familial hypercholesterolemia

## Abstract

Glycosylation, a post-translational modification, plays a crucial role in proper localization and function of proteins. It is regulated by multiple glycosyltransferases and can be influenced by various factors. Inherited missense mutations in glycosylated proteins such as NOTCH3, Low-density lipoprotein receptor (LDLR), and Amyloid precursor protein (APP) could affect their glycosylation states, leading to cerebral small vessel disease, hypercholesterolemia, and Alzheimer’s disease, respectively. Additionally, physiological states and aging-related conditions can affect the expression levels of glycosyltransferases. However, the interplay between mutations in glycosylated proteins and changes in their glycosylation levels remains poorly understood. This mini-review summarizes the effects of glycosylation on transmembrane proteins with pathogenic mutations, including NOTCH3, LDLR, and APP. We highlight the synergistic contributions of missense amino acids in the mutant proteins and alterations in their glycosylation states to their molecular pathogenesis.

## 1 Introduction

Glycosylation, a post-translational modification involving the addition of sugar chains to proteins, is categorized into *N-* and *O-*linked glycosylation. This process regulates protein folding, processing, transport, function, interactions, and turnover. Various glycosyltransferases are responsible for transferring glycans to proteins.

Abnormal glycosylation, driven by multiple factors, can lead to numerous pathogenic processes. The primary genetic pathology associated with aberrant glycosylation is congenital disorders of glycosylation (CDG). CDG involves loss-of-function mutations in genes related to the synthesis and transfer of glycan, often leading to developmental and neurological diseases (Reily et al., 2019). In contrast, some missense mutations in genes encoding glycosylated proteins, such as transmembrane proteins, can affect their glycosylation states and lead to pathogenesis. For example, missense mutations in NOTCH3 alter its *O-*glycosylation states and lead to cerebral autosomal dominant arteriopathy with subcortical infarcts and leukoencephalopathy (CADASIL) (Joutel et al., 1996; Arboleda-Velasquez et al., 2005; Suzuki et al., 2024). In addition, Low-density lipoprotein receptor (LDLR) and Amyloid precursor protein (APP) are also glycosylated proteins and their glycosylation levels could be affected by mutations related with inherited diseases, familial hypercholesterolemia (FH) and familial Alzheimer’s disease (FAD), respectively (Akasaka-Manya et al., 2008; Pedersen et al., 2014; Akasaka-Manya and Manya, 2020). Physiological aspects also influence glycosylation states. Expression levels of glycosyltransferases vary among different tissues and cell types (Indellicato and Trinchera, 2021; Sobral et al., 2022). Furthermore, degenerative conditions, such as aging, can alter the expression of glycosyltransferases (Vanhooren et al., 2011; Oinam et al., 2020; Sobral et al., 2022), the amounts of sugar-nucleotides as glycan sources (Imae et al., 2024), and thereby protein glycosylation states (Sato and Endo, 2010; Itakura et al., 2016; Gudelj et al., 2018; Zhang et al., 2024). However, the relationship between missense mutations in glycosylated proteins and changes in their glycosylation states is not fully understood.

This mini-review summarizes how mutations and glycosylation in the transmembrane proteins contribute to the pathogenesis, with a focus on NOTCH3, Low-density lipoprotein receptor (LDLR), and Amyloid precursor protein (APP). These examples highlight the synergistic roles of missense amino acids and the protein glycosylation changes in disease progression.

## 2 NOTCH3 and cerebral autosomal dominant arteriopathy with subcortical infarcts and leukoencephalopathy (CADASIL)

### 2.1 Effects of glycosylation on NOTCH3 functions and proteostasis

**FIGURE 1:**
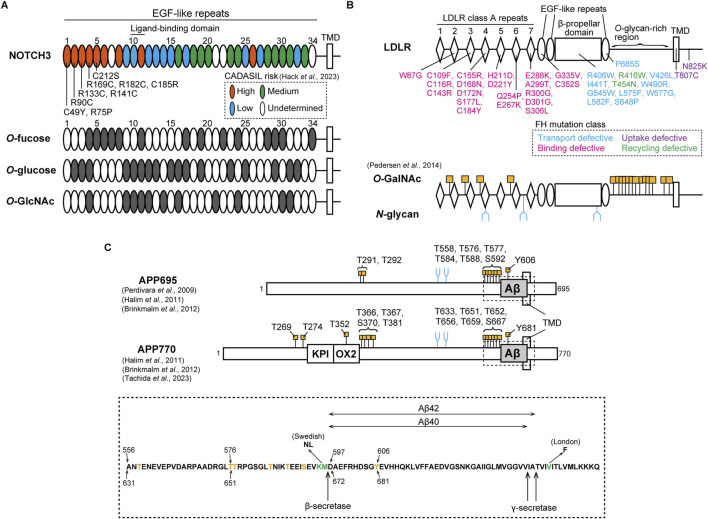
Pathological mutation and glycosylation amino acid sites of NOTCH3, LDLR, and APP. **(A)** Upper: NOTCH3 consists of 34 EGF-like repeats in its ectodomain, as shown in an oval. The ligand-binding domain exists in EGF-like repeats 10 and 11. EGF-like repeats at which cysteine-altering mutations are associated with high, medium, low and undetermined risks for CADASIL are shown in orange, green, cyan, and white, respectively, as classified in a recent study (Hack et al., 2023). Representative pathogenic mutations in EGF-like repeats 1 to 5 are shown. TMD represents transmembrane domain. Below: EGF-like repeats that harbor the consensus sites for *O-*fucose (C^2^XXXXS/TC^3^), *O-*glucose (C^1^XSXA/PC^2^ and C^3^XNTXGSFXC^4^), and *O-*GlcNAc (C^5^XXGY/FS/TGXXXC^6^ and C^5^XXGY/FS/TGXXC^6^) are shown in gray. **(B)** Upper: LDLR consists of 7 class A repeats with the linker region, a β-propeller domain surrounded by two and one EGF-like repeats, an *O-*glycan-rich domain, a transmembrane domain, and an intracellular domain. The ligand-binding domain exists in the class A repeats. FH-causing missense mutations are shown in cyan, magenta, purple, and green, depending on functional phenotypes with defects in transport, binding, uptake, and recycling, respectively. TMD represents transmembrane domain. Below: *O-*GalNAc and *N-*glycan modification sites in the LDLR as identified by Pedersen et al. (2014). *O-*GalNAc is modified at four consensus sites, XX-C^6^-XXXT-C^1^-XX, within linker regions between the class A repeats and at multiple sites of the *O-*glycan-rich domain, as shown in orange. *N-*glycan is also attached to three sites in the class A repeats and the β-propeller domain, as represented in blue. **(C)** Upper: APP isoforms, APP695 and APP770. Compared to APP695, APP770 has KPI and OX2 domains. Aβ, as shown in gray, is cleaved by β- and γ-secretase. *O-*GalNAc and *N-*glycan are attached to specific sites, as shown in orange and blue, respectively (Perdivara et al., 2009; Halim et al., 2011; Brinkmalm et al., 2012; Tachida et al., 2023). TMD represents the transmembrane domain. Below: amino acid sequences including the Aβ region, as highlighted in the upper figure by a dotted box. Threonine, serine, and tyrosine residues modified with *O-*GalNAc are shown in orange. The amino acids shown in green are mutated sites observed in FAD Swedish and London families. The amino acid positions for APP695 and APP770 are described at the upper and bottom sides, respectively.

The *NOTCH3* gene encodes a single transmembrane receptor involved primarily in differentiation and survival of vascular smooth muscle cells (VSMCs) and pericytes in the adult vasculature (Domenga et al., 2004; Liu et al., 2010; Wang et al., 2014; Henshall et al., 2015; Vanlandewijck et al., 2018). NOTCH3 is one of NOTCH family (NOTCH1-4) and features 34 epidermal growth factor (EGF)-like repeats in its extracellular domain, including binding sites for canonical Notch ligands, such as Jagged (JAG)-1 and −2, and Delta-like ligands (DLL)-1 and −4 (Figure 1A) (Bray, 2006; Bray, 2016). The interaction between the NOTCH receptors and ligands triggers the dissociation of the extracellular and intracellular domain, which induces the upregulation of downstream gene expression (Nichols et al., 2007; Meloty-Kapella et al., 2012). The NOTCH3 ectodomain is endocytosed by JAG1 and DLL4 (Watanabe et al., 2011; Suzuki et al., 2021; Suzuki et al., 2024). In each EGF-like repeat of NOTCH, three disulfide bonds form between C^1^-C^3^, C^2^-C^4^ and C^5^-C^6^, where the number on cysteine denote their position within an EGF-like repeat. Moreover, NOTCH EGF-like repeats can be modulated by *O-*glycosylation, such as *O-*fucose, *O-*glucose, and *O-N-*acetylglucosamine (*O-*GlcNAc), which are modified by Protein *O-*fucosyltransferase 1 (POFUT1) (Moloney et al., 2000; Wang et al., 2001; Stahl et al., 2008), Protein *O-*glucosyltransferase 1-3 (POGLUT1-3) (Wang et al., 2001; Acar et al., 2008; Takeuchi et al., 2018), and EGF domain-specific *O-*linked *N-*acetylglucosamine transferase (EOGT) (Matsuura et al., 2008; Sakaidani et al., 2012), respectively (Figure 1A). The *O-*glycans are linked to serine or threonine within the cysteine-flanked consensus sequences of EGF-like repeats. Specifically, *O-*fucose attaches at C^2^XXXXS/TC^3^, *O-*glucose at C^1^XSXA/PC^2^ and C^3^XNTXGSFXC^4^, and *O-*GlcNAc at C^5^XXGY/FS/TGXXXC^6^ and C^5^XXGY/FS/TGX XC^6^ (Varshney and Stanley, 2018; Urata and Takeuchi, 2019). The POFUT1-mediated *O-*fucose modification regulates the trafficking and binding ability of Notch receptors (Okamura and Saga, 2008; Stahl et al., 2008). POFUT1 also functions as a glycosyltransferase-independent chaperone, helping Notch folding (Okajima et al., 2005). Monosaccharide *O-*fucose can be elongated to a tetrasaccharide with GlcNAc, galactose, and neuraminic acid, which are added by β-1,3*-N-*acetylglucosaminyltranseferase (Fringe), β-4-galactosyltranseferase, and sialyltransferase, respectively (Brückner et al., 2000; Moloney et al., 2000). Fringe modulates ligand-binding ability and signaling activity (Brückner et al., 2000; Moloney et al., 2000; Yang et al., 2005; Taylor et al., 2014; Kakuda and Haltiwanger, 2017; Kakuda et al., 2020; Suzuki et al., 2024). There are three homologs in mammals: Lunatic fringe (LFNG), Manic fringe (MFNG), and Radical fringe (RFNG), which glycosylate NOTCH at common and unique sets of sites. LFNG and MFNG inhibit the JAG1-dependent activity of NOTCH1, but RFNG enhances it (Moloney et al., 2000; Kakuda and Haltiwanger, 2017). Among three homologs, our recent study has demonstrated that a human pericyte cell line expresses RFNG at a higher level compared to LFNG and MFNG, and long-term culture-induced senescence increases RFNG in pericytes (Suzuki et al., 2024). Overexpression of RFNG in a NOTCH3-expressing HeLa cell line impairs JAG1-NOTCH3 but enhances DLL4-NOTCH3 activity (Suzuki et al., 2024). These findings suggest that RFNG can regulate the NOTCH3 function and turnover under cerebral microenvironment and aging conditions. Moreover, given that *O-*glucose and *O-*GlcNAc regulates the folding and transport of NOTCH1 (Takeuchi et al., 2017; Ogawa et al., 2020; Zhang et al., 2022), the three types of *O-*glycan may cooperatively play roles in NOTCH3 signaling function and proteostasis.

### 2.2 Roles of glycosylation in CADASIL pathogenesis

Mutations in *NOTCH3* gene cause Cerebral Autosomal Dominant Arteriopathy with Subcortical Infarcts and Leukoencephalopathy (CADASIL), a cerebral small vessel disease marked by recurrent ischemic strokes and vascular dementia (Joutel et al., 1996; Yamamoto et al., 2023; Mizuta et al., 2024). The disease is characterized by the degeneration of mural cells and the abnormal deposition of granular osmiophilic material (GOM), containing NOTCH3 protein as a main component (Ruchoux et al., 1995; Mayer et al., 1999; Joutel et al., 2000; Ishiko et al., 2006; Tikka et al., 2009; Lewandowska et al., 2011; Morroni et al., 2013). Based on investigations with disease model animals, the pathological features of CADASIL appear in an age-dependent manner (Joutel et al., 2010; Ghosh et al., 2015; Gravesteijn et al., 2019). High-risk pathogenic mutations are clustered within the EGF-like repeats 1 to 6, which are not ligand-binding domain (Rutten et al., 2016; Hack et al., 2023) (Figure 1A). Furthermore, most CADASIL mutations alter cysteine residues, inducing aggregation. NOTCH3 C49Y, R90C, R133C, R141C, and C183R mutations result in the multimerization of the EGF-like repeats 1-3 and 1-5 fragmented protein due to abnormal disulfide bond formation (Opherk et al., 2009; Duering et al., 2011; Young et al., 2022). Despite the tendency of NOTCH3 R133C and C185R to accumulate within the endoplasmic reticulum and at the cell membrane (Takahashi et al., 2010; Watanabe et al., 2011; Suzuki et al., 2024), our research indicates that this accumulation is influenced by the cellular environment as well as the mutation itself (Suzuki et al., 2021). In addition, signaling defects in NOTCH3 mutants might also play a role in disease pathogenesis, which remains a topic of debate (Joutel et al., 2004; Peters et al., 2004; Jin et al., 2008; Dupré et al., 2024; Romay et al., 2024). Taking all factors into account, the cellular accumulation and signaling defects of NOTCH3 CADASIL mutant proteins may be contingent on cellular conditions, including the glycosylation states of NOTCH3. Since the *O-*glycan consensus sequences of NOTCH include cysteine residues at defined positions, numerous pathogenic cysteine-altering mutations can change NOTCH3 glycosylation states, leading to abnormal function and turnover. POFUT1 does not facilitate the folding of a CADASIL-like *Drosophila* Notch mutant C599Y, which corresponds to C542Y in human NOTCH3, in comparison to the wild-type Notch (Okajima et al., 2005). Mouse NOTCH3 CADASIL-like mutants R91C, R170C, and C213S (corresponding to R90C, R169C, and C212S in human NOTCH3, respectively), exhibit impairment of LFNG-mediated modification in the NOTCH3 EGF-like domains 1 to 5 (Arboleda-Velasquez et al., 2005). LFNG tends to increase the aggregation propensity of NOTCH3 C185R but not that of NOTCH3 WT (Suzuki et al., 2021). Our recent study has demonstrated that an increase in RFNG expression leads to a greater reduction in signaling activity and turnover of NOTCH3 R141C and C185R compared to the WT (Suzuki et al., 2024). Furthermore, LC-MS/MS analysis demonstrated that RFNG modifies NOTCH3 C185R at a lesser extent to the WT in the EGF-like repeat 4 but to a similar extent in the EGF-like repeat-11 (Suzuki et al., 2024). Given that specific *O-*fucose sites on NOTCH1 play unique roles in how Fringe modulates NOTCH1 activity through *O-*fucose elongation (Kakuda and Haltiwanger, 2017), reduced RFNG-mediated glycosylation in NOTCH3 C185R EGF-like repeat 4, compared to NOTCH3 WT, may lead to impaired signaling activity and increased resistance to degradation of NOTCH3 C185R. Furthermore, investigating the NOTCH3 EGF-like domains linked to CADASIL risk and their glycosylation consensus sites may explain the high risk for CADASIL (57) (Figure 1A). For example, patients with NOTCH3 R182C mutation in the EGF-like domain 4 have a higher accumulation of NOTCH3 protein around the vessels compared to those with R141C in EGF-like repeat 3 and R75P in EGF-like repeat 1 (Ishiyama et al., 2024). This may potentially be attributed to the unique positioning of the EGF-like domain 4, which has three types of *O-*glycosylation consensus amino acid sequences (Figure 1A). On the other hand, the modification of NOTCH1 EGF-like domains by Fringe varies with cell type (Matsumoto et al., 2022). Therefore, comprehensive analyses are needed to investigate the *O-*glycosylation states of NOTCH3 in specific cell types, such as vascular muscle cells and pericytes, as well as under senescent conditions.

## 3 Low-density lipoprotein receptor (LDLR) and familial hypercholesterolemia (FH)

### 3.1 Roles of glycosylation on LDLR functions and transport

The low-density lipoprotein receptor (LDLR) is a transmembrane receptor expressed in many types of cells, regulating plasma cholesterol levels (Goldstein and Brown, 1985). The extracellular region at the N-terminal domain harbors seven LDLR class A repeats, each of which is a cysteine-rich module with three disulfide bonds. Additionally, there are three EGF-like repeats along with a β-propeller domain and clustered *O-*glycan-rich sites (Figure 1B). LDLR-mediated cholesterol maintenance begins with LDLR binding to plasma LDL, a circulating cholesterol carrier containing apolipoprotein families such as apolipoprotein B (APOB), and subsequently undergoing clathrin-mediated endocytosis (Goldstein and Brown, 1985; Esser et al., 1988; Russell et al., 1989). The binding of LDLR to LDL involves the LDLR class A repeats 1-7, among which deletion of class A repeat-5 impacts LDLR binding ability the most (Russell et al., 1989). After transport to endosomes, the acidic conditions enable the EGF-like repeats and β-propeller domains of LDLR to change conformation (Davis et al., 1987; Klee and Zimmermann, 2019). This results in the dissociation of LDLR-LDL complex and subsequent recycling of LDLR to the cell membrane (Davis et al., 1987). LDLR is known to be modified with both *O-* and *N-*glycans (Cummings et al., 1983; Pedersen et al., 2014) (Figure 1B). Defects in producing UDP-*N-*acetylgalactosamine (GalNAc) lead to reduced expression of LDLR at the cell membrane, suggesting that mucin-type *O-*GalNAc modification of LDLR is required for the stabilization (Kozarsky et al., 1988). Mass spectrometric analysis showed that the linker domains between the class A repeats are modified with *O-*GalNAc at threonine residues in the consensus sequence XX-C^6^-XXXT-C^1^-XX (Figure 1B) (Pedersen et al., 2014). Among the polypeptide *N-*acetylgalactosaminyltransferase family (ppGalNAc-Ts), ppGalNAc-T11 mainly regulates *O-*GalNAc modification in LDLR (Pedersen et al., 2014). Furthermore, the *O-*GalNAc in the linker regions regulates LDLR affinity for LDL (Wang et al., 2018). In contrast, the *O-*glycan-rich region stabilizes the protein structure but does not affect binding affinity for LDL (Davis et al., 1986a). LDLR class A repeats and β-propeller domain are also modified with *N-*glycans (Figure 1B), but their functions remain unclear (Pedersen et al., 2014).

### 3.2 Relevance of glycosylation with FH pathogenesis

*LDLR* variants are associated with familial hypercholesterolemia (FH), leading to arteriosclerosis and myocardial infarction (Goldstein and Brown, 1985). Aging plays a key role in causing coronary heart disease in FH (Mabuchi et al., 1989; Perak et al., 2016). Although FH pathogenic mutations are widely reported in *LDLR* without a hot spot (Vrablik et al., 2020), FH mutations in the class A repeat 5 are abundant and lead to higher LDL-cholesterol levels in blood than those in other domains (Gudnason et al., 1994; Day et al., 1997). Based on structural and functional analysis, the mutations are classified into classes 1 to 5 (Tolleshaug et al., 1983; Etxebarria et al., 2015; Benito-Vicente et al., 2018; Banerjee et al., 2019; Alves et al., 2021; Graça et al., 2022). Class 1: the null alleles do not produce LDLR, resulting in no visible protein bands on polyacrylamide gel electrophoresis (Tolleshaug et al., 1983). Class 2: transport-defective alleles express the glycosylation-immature form due to retention in the endoplasmic reticulum (Tolleshaug et al., 1983; Etxebarria et al., 2015; Benito-Vicente et al., 2018; Banerjee et al., 2019; Kizhakkedath et al., 2019; Alves et al., 2021; Graça et al., 2022). Class 3: binding-deficient alleles produce mature LDLR transported to the cell membrane, but impair its binding ability to LDL (Tolleshaug et al., 1983; Etxebarria et al., 2015; Benito-Vicente et al., 2018; Banerjee et al., 2019; Alves et al., 2021; Graça et al., 2022). Class 4: internalization-deficient alleles exhibit low endocytosis of LDLR (Lehrman et al., 1985; Davis et al., 1986b; Benito-Vicente et al., 2018). Class 5: recycling-defective mutations in the β-propeller domain impair the recycling pathway after the dissociation of the LDLR-LDL complex (Benito-Vicente et al., 2018; Banerjee et al., 2019). While the LDLR variants affect 55% of amino acids located within five or fewer residues from glycosylation sites (Klee and Zimmermann, 2019), the precise mechanisms by which the missense mutations in LDLR affect its glycosylation, thereby leading to insufficient binding, uptake, and recycling phenotypes, remain unclear. One possibility is that cysteine-altering mutations could affect the structure of the consensus *O-*GalNAc amino acid sequence in the class A linker regions. This may result in reduced *O-*GalNAc modification and thereby less binding ability of LDLR (Figure 1B) (Wang et al., 2018). Cysteine-sparing mutations in *LDLR* may also cause abnormal disulfide bonds, as seen in mutations in *NOTCH3,* and subsequently reduced glycosylation (Wollenweber et al., 2015). Additionally, aging-mediated expression changes in ppGalNAc-Ts and mutations in *LDLR* may cooperatively contribute to pathogenesis (Sobral et al., 2022). Comprehensive investigation of glycosylation in LDLR mutants, under conditions with aging and various cell types, may help in understanding of the glycosylation-mediated mechanisms underlying FH pathogenesis.

## 4 Amyloid precursor protein (APP) and familial Alzheimer’s disease (FAD)

### 4.1 Effects of glycosylation on APP transport and processing

The amyloid precursor protein (APP) is a transmembrane protein involved in Alzheimer’s disease through the production of amyloid-β (Aβ). APP exists in three isoforms: APP695, APP751, and APP770. APP695 is predominantly expressed in mature neurons, while APP770 is expressed in brain endothelial cells (Wertkin et al., 1993; Kitazume et al., 2010). APP undergoes amyloidogenic and non-amyloidogenic pathways, regulated by different processing enzymes. The amyloidogenic process initiates with cleavage by β-secretase 1 (BACE1) in the Goldi complex and endosome, leading to the release of the toxic peptide Aβ through subsequent processing by γ-secretase. In the non-amyloidogenic pathway, APP is cleaved by α-secretase and γ-secretase, producing the extracellular soluble APP-α protein. These distinct processing pathways are regulated by *N-* and *O-*linked glycosylation attached to APP (Figure 1C) (Akasaka-Manya and Manya, 2020). APP has two *N-*glycosylation sites, and APP695 mutants deficient in *N-*glycosylation show impaired transport to the cell membrane (Yazaki et al., 1996; Tsatsanis et al., 2019). β-galactoside α2,6-sialyltransferase (ST6Gal-I) adds sialic acid to the terminal galactose of *N-*linked glycans. Overexpression of ST6Gal-I enhances both α-cleavage and β-cleavage of APP695 by increasing its sialylation, resulting in elevated Aβ release (Nakagawa et al., 2006). The bisecting GlcNAc, a form of *N-*linked glycan, is modified by GnT-III. Overexpression of GnT-III reduces Aβ secretion in Neuro2a cells through increased glycosylation of APP695 and BACE1, indicating that increased bisecting GlcNAc on APP695 and BACE1 is protective against Aβ production (Akasaka-Manya et al., 2010). Mass spectrometry analyses show that APP has threonine and serine sites modified with *O-*GalNAc glycans (Perdivara et al., 2009; Halim et al., 2011; Brinkmalm et al., 2012; Tachida et al., 2023) (Figure 1C). The *O-*glycan-deficient mutation in APP695 causes retention in the endoplasmic reticulum, impairing the processing pathways (Tomita et al., 1998). ppGalNAc-T6 glycosylates APP695 at T577, promoting α-cleavage but not β-cleavage. This results in decreased Aβ release (Akasaka-Manya et al., 2017). ppGalNAc-T2 also regulates Aβ production from APP695 (Liu et al., 2017) and APP770 (Tachida et al., 2023). Moreover, Aβ peptide purified from human cerebrospinal fluid, which may be derived from all APP isoforms, contains the unique *O-*GalNAc modification at the tyrosine residue, APP695 Y606 and APP770 Y681, respectively (Halim et al., 2011; Brinkmalm et al., 2012). This modification regulates the secondary structure and processing efficiency of the APP fragment (Singh et al., 2021). Similar to the Notch protein, the extracellular domain of APP695 could be modified with *O-*GlcNAc at the threonine residues, T291, T292, and T576 (Chun et al., 2017). *O-*GlcNAcase inhibition with PUGNAc, which enhances *O-*GlcNAcylation on APP695, reduces Aβ release by decreasing APP695 internalization from the cell membrane (Jacobsen and Iverfeldt, 2011; Chun et al., 2015). The glycosyltransferase for APP695 *O-*GlcNAcylation has not been identified yet, but EOGT could play a role (Akasaka-Manya and Manya, 2020). On the other hand, the effects of *O-*glycosylation on processing and transport differ between APP695 and APP770 (Tachida et al., 2023). Accordingly, the diverse *N-* and *O-*glycosylation at multiple amino acid sites regulate the folding, cellular localization, and cleavage pathways of APP695 and APP770 in distinct ways, thereby controlling Aβ release.

### 4.2 Impacts of glycosylation on Aβ production from APP pathogenic mutants

Mutations in the *APP* gene cause familial Alzheimer’s disease (FAD). The APP695 K595N and M596L mutations, known as the Swedish mutation, exhibit higher cleavage efficiency by BACE1 compared to wild-type APP, leading to increased Aβ production (Citron et al., 1992; Perez et al., 1996; Thinakaran et al., 1996). APP produces different Aβ forms, Aβ40 and Aβ42, with the latter being less soluble (Burdick et al., 1992; Jarrett et al., 1993). The APP695 London-type mutation, V642F, located near the γ-secretase site, increases Aβ42 release but not Aβ40 (Suzuki et al., 1994). APP695 Swedish and London-type mutants exhibit increased bisecting GlcNAc and core fucose in *N-*glycans (Akasaka-Manya et al., 2008). Although bisecting GlcNAc protects against Aβ production in APP WT (Akasaka-Manya et al., 2010), the effects of enhanced *N-*glycosylation of APP Swedish and London mutants on additional Aβ production remain unknown. BACE1 can directly reduce ST6Gal-I activity through its cleavage, which competes with APP695 WT as a BACE1 substrate (Kitazume et al., 2001). However, the APP695 Swedish mutant cannot reduce ST6Gal-I activity compared to APP WT, potentially increasing Aβ production due to enhanced sialylation (Kitazume et al., 2001; Nakagawa et al., 2006). Furthermore, APP770 Y681 *O-*GalNAcylation increases β-cleavage of the Swedish mutant but not that of WT, indicating the synergistic contributions of APP Swedish mutation and the *O-*GalNAc modification at the tyrosine to Aβ production (Singh et al., 2021). Therefore, *N-*glycan and *O-*glycosylation can impact Aβ production from APP FAD mutant proteins, in addition to the effects of mutations themselves. Future work is required to determine the glycosylation states of APP FAD mutants in different cell types such as neurons and cerebral endothelial cells.

## 5 Summary and conclusion

**FIGURE 2:**
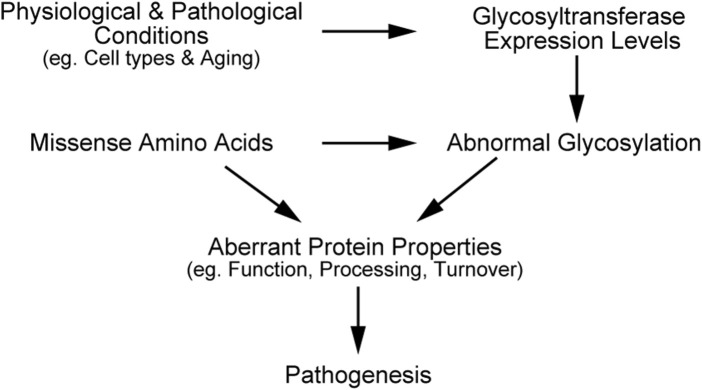
Schematic illustration explaining synergistic effects of mutation and glycosylation on disease progression.

In this review, we have outlined the impacts of *O-* and *N-*linked glycosylation on the function, processing, and turnover of pathogenic mutants, including NOTCH3, LDLR, and APP. Missense mutations in the transmembrane proteins can alter their glycosylation states, partly due to structural disturbances around glycosylated sites. The variants can change the protein localization of proteins, resulting in glycosylation pattern that differ from the wild-type. Moreover, cell types and aging are known to affect expression levels of glycosyltransferases such as radical fringe and ppGalNAc-Ts (Sobral et al., 2022; Suzuki et al., 2024), and consequently change the glycosylation levels of NOTCH3, LDLR, and APP. Therefore, missense amino acids, physiological and pathological changes of glycosyltransferases expression, and abnormal glycosylation in mutant proteins can synergistically contribute to pathogenesis (Figure 2). Employing state-of-the-art techniques to detect *N-* and *O-*glycosylation and investigating the combined effects of glycosylation and mutations could enhance our understanding of molecular pathogenic mechanisms. Meanwhile, it is crucial to determine the glycosylation states of mutant proteins with an appropriate experimental design, considering both physiological and pathogenic conditions.
